# Dietary Saturated Fat and Bone Health in Young Adults: The Young Finns Cohort

**DOI:** 10.1007/s00223-022-01008-5

**Published:** 2022-07-27

**Authors:** S. Tolonen, M. Juonala, M. Fogelholm, K. Pahkala, M. Laaksonen, M. Kähönen, H. Sievänen, J. Viikari, O. Raitakari

**Affiliations:** 1 Research Centre of Applied and Preventive Cardiovascular Medicine, University of Turku, Turku, Finland; 2 Department of Medicine, University of Turku and Turku University Hospital, Turku, Finland; 3 Murdoch Children’s Research Institute, Parkville, VIC Australia; 4 Department of Food and Nutrition, University of Helsinki, Helsinki, Finland; 5 Centre for Population Health Research, University of Turku and Turku University Hospital, Turku, Finland; 6 Department of Clinical Physiology, Tampere University Hospital, Tampere, Finland; 7 Faculty of Medicine and Health Technology, Tampere University, Tampere, Finland; 8 The UKK Institute for Health Promotion Research, Tampere, Finland; 9 Department of Clinical Physiology and Nuclear Medicine, Turku University Hospital, Turku, Finland

**Keywords:** Quantitative computed tomography (QCT), Quantitative ultrasound (QUS), Bone fracture, Dietary saturated fat

## Abstract

Previous studies suggest that saturated fat (SFA) intake may negatively impact on bone. However, few human studies on the topic exist. Women and men aged 31–46 years from the Cardiovascular Risk in Young Finns study attended the peripheral quantitative computed tomography and ultrasound bone measurements in 2008 (*n* = 1884–1953, ~ 56% women). In addition, fracture diagnoses in 1980–2018 were searched for the national health care registers and 431 participants had at least one fracture. Food consumption was gathered with the 48-h dietary recall interviews and food frequency questionnaire in 1980–2007. In the present study, radial, tibial, and calcaneal bone traits, and fractures were examined relative to the long-term intake of SFA. No consistent associations were seen between bone outcomes and SFA intake that would have replicated in both women and men. The only evidence for differential distributions was seen in cortical density and cortical-to-total area ratio at the radial shaft, and speed of sound at the calcaneus, which were 0.1–0.4% higher in women in the lowest tertile of SFA intake compared with the highest tertile. In addition, among men, the odds ratio (OR) of fractures was greater in the second (OR 1.86, 95% confidence interval (CI) 1.03–3.33) and third tertile of SFA intake (OR 2.45, 95% CI 1.38–4.36) compared with the lowest tertile, independently of many risk factors of osteoporosis. In this observational study, we found no robust evidence of the associations of dietary long-term SFA intake with bone outcomes. Therefore, additional studies are needed to confirm the association of dietary SFA with bone health in humans.

## Introduction

A decrease in bone mineral density (BMD) and content (BMC), or a change in bone architecture, may subsequently lead to decreased bone strength and eventually also to osteoporosis [1]. A general diagnostic criterion of osteoporosis is defined as a BMD 2.5 standard deviation or more below the mean of young adult women. Osteoporotic bones break more likely than a healthy bone and may cause severe disability to their carrier including, for instance, pain and loss of independence. It is estimated that in the European Union, the annual number of incident fractures was approximately 3.5 million in 2010 and was estimated to rise to 4.5 million until the year 2025 [2].

There is an experimental evidence that dietary fats may affect bone tissue as, for instance, shown in a study conducted in mice where a diet including plenty of saturated fat was associated with lower total bone mineral mass and density [3]. These mice have probably been influenced by dyslipidemia which has shown to inhibit bone formation and increase bone resorption [4, 5]. The delivery of cholesterol to osteoclasts by low-density lipoprotein (LDL) increases osteoclast activity and formation; meanwhile, the efflux of cholesterol from osteoclasts to high-density lipoprotein (HDL) induces osteoclast apoptosis by suppressing intracellular signals and osteoclast fusion. Other factors in the cholesterol pathway mentioned regulating osteoclastogenesis were, for instance, estrogen-related receptor α, oxysterol, liver X receptors, and retinoid X receptors α and β [5]. Also, the growth and function of osteoblasts require LDL and other lipid particles as an energy source which acquisition and oxidation are regulated by several factors such as those related to the Wnt and parathyroid hormone signaling pathways [4].

Fatty acids, both endogenous and exogenous, may also connect with bone cells directly and thereby influence bone remodeling and structure [5, 6]. The effects of different fatty acids in targeted bone cells are mediated through several receptors in which fatty acids can attach and thereby influence internal signaling cascades such as osteoclast stimulating receptor activator of nuclear factor kappa-B ligand (RANKL) pathway [6]. In addition to bone cells, Bao et al. noted that fatty acids can also influence other properties of bone, such as calcium metabolism and bone collagen synthesis. In human cell studies, unsaturated fatty acids have been found to suppress the formation and activity of osteoclasts [7, 8]. In addition, the intakes of polyunsaturated fatty acids and fish have been found to be positively associated with BMD at the hip and lumbar spine and the lower risk of fractures mainly in older adults [9, 10]. Contrary to unsaturated fatty acids, saturated fatty acids (SFA), such as palmitic acid, have been noticed to enhance the formation and survival of murine osteoclasts and influence negatively on femoral cortical density [3, 11, 12]. In addition to enhanced bone resorption activity, SFA may also inhibit bone formation as shown earlier in vitro [13]. In the previous observational and case–control studies, SFA intake was found to be associated with lower BMD and increased risk of fractures at the hip [14–17]. However, the intake of saturated fat may also have some protective effect on femoral neck bone density as shown in a population-based study using the Healthy Eating Index based on Dietary Guidelines for Americans [18].

Associations of fatty acids and bone may also be related to the insulin-induced uptake of glucose and fatty acids in the bone marrow. Ojala et al. noticed that men with insulin resistance had the decreased uptake of fatty acids and glucose in bone marrows and lower bone turnover markers in plasma compared with controls [19]. Weivoda et al. revealed that dipeptidyl peptidase-4 secreted by osteoclasts might be a link between bone and energy metabolism as it blocks the effect of glucagon-like peptide-1 on the pancreas and thereby reduces the synthesis of insulin and its secretion to circulation [20]. In addition to unbalanced glucose homeostasis, impaired bone marrow metabolism has been found in conditions related to remarkable overweight and underweight [21]. In general, impaired bone marrow metabolism may lead to increased adiposity inside the bone which, on the other hand, seems to be inversely associated with BMD and in some studies related to the prevalence of vertebral fractures [21]. Previously, factors that have been found regulating the association of bone marrow adiposity and bone are, for instance, insulin-like growth factor-1 [22, 23], estrogen [24, 25], sclerostin [26, 27], and proinflammatory cytokines and adipokines [28, 29].

In general, the intake of SFA is above the recommendations in many western populations, such as those in Europe and North America, and therefore, it is important to study the associations of SFA with human health. In the present study population of adult women and men, we investigated the associations of dietary SFA with different bone phenotypes at calcaneus, tibia, and radius. We also studied whether SFA was associated with bone fractures in the present population. To our knowledge, no other previous study has as long follow-up data on saturated fat intake as here which allows us to study if the mean long-term intake of saturated fat accumulated between 1980 and 2007 will have an impact on bone outcomes studied.

## Methods

### Study Population and Bone Data

The Cardiovascular Risk in Young Finns Study (the Young Finns cohort) explores the contribution of childhood factors to the risk of diseases in adulthood [30]. In 1980, the first baseline study was carried out by the universities and university hospitals of Turku, Helsinki, Tampere, Kuopio, and Oulu (3–18-years, *n* = 3596). After this, there have been several follow-ups of which the latest was conducted in 2018–2020. The participation rate has varied from 83.2% at the first survey to around 60–80% at the later follow-ups. In 2008, persons in the register of the Young Finns cohort received an invitation to the peripheral quantitative computed tomography (pQCT) and quantitative ultrasound (QUS) bone measurements [31]. Altogether, 1884 participants aged 31–46-years (of which 56.2% women) from Turku, Helsinki, Tampere, Kuopio, and Oulu attended the pQCT measurements. The corresponding number of the QUS measurements was 1953 (56% women).

The non-weight-bearing radius and weight-bearing tibia were measured with the same pQCT measurement device in each study center (XCT 2000R, Stratec Medizintechnik GmbH, Pforzheim, Germany). In ~ 99.7% of the cases, radius was measured on the nonwriting hand and tibia on the left leg if no contraindication emerged. The lengths of ulna and tibia were measured with a tape measure and the measurement sections of the distal radius and tibia were defined as 4% and 5% from the distal cortical endplate, respectively. The diaphyseal sites were 30% for both studied bones. Finally, 1842–1856 radius and 1853–1857 tibia measurements were successfully scanned and analyzed in the distal and shaft parts of the bones, respectively (~ 98% of those who participated). Numbers for the successful ultrasound scans of the left heel were 1494–1515 (~ 77% of the participants).

### Bone Geometrical Traits and Mineral Density

The analysis of bone density and geometrical traits from the pQCT scan images was done using specific threshold values and mode options according to the previously described standard procedures [32]. Analyses of the pQCT measurements yielded the following bone parameters: BMC (mg), trabecular and cortical bone mineral densities (mg/cm^3^), and total and cortical bone areas (mm^2^). Additionally, the following bone strength indices were calculated: stress–strain index (SSI, mm^3^) and cortical-to-total area ratio. SSI reflects the torsional strength of the bone diaphysis and is based on the density-weighted polar section modulus.

At the calcaneus, the speed of sound (SOS, m/s) and broadband ultrasound attenuation (BUA, dB/MHz) were measured with the QUS technique (Sahara Clinical Bone Sonometer, Hologic Inc., Waltham, MA, USA). The in vivo precision of the pQCT and QUS measurements evaluated in a subgroup (*n* = 39) varied between 0.3 and 4.8%.

### Fractures

Primary and secondary fracture diagnosis codes of the skull, cervical vertebrae, ribs, sternum, thoracic vertebrae, lumbar vertebrae, pelvis, shoulder girdle, upper arm, forearm, carpal and metacarpal bones, phalanges of hand and foot, femur, patella, lower leg, and foot in 1980–2018 were searched for the national health care registers maintained by the Finnish Institute for Health and Welfare according to the International Classification of Diseases 9th and 10th revisions (ICD-9 and ICD-10) with the SPSS-software. In total, there were 431 participants with at least one registered fracture diagnosis.

### Dietary Habits

At the baseline in 1980, dietary data were gathered with 48-h dietary recall interviews done by trained dietitians for around half of the study subjects (3–18 years, *n* = 1768). The interviews were repeated in 1986 and 2001 with participation rates of 59–68% [33]. This method gives detailed information on participants’ dietary habits during the two days prior to the interview. In 2007, information on food consumption and nutrient intake was collected with a modified 131- item food frequency questionnaire developed by the Finnish Institute for Health and Welfare [34]. In 1980 and 1986, the nutrient calculations were based on the Finnish food composition tables maintained by the University of Helsinki and analytical data were obtained from the local food industry. Later, 2001 and 2007, the nutrient contents of reported foods were calculated using the Finnish Food Composition database, Fineli® [35]. Dietary intakes of energy (kJ/day), carbohydrate (E%), fiber (g/day), protein (E%), total fat (E%), polyunsaturated fat (E%, g/day), monounsaturated fat (E%, g/day), calcium (mg/day), vitamin D (μg/day), and dairy products (g/day) were given as a mean intake/day or as an energy percentage of total energy intake. In addition, the intakes of dietary SFA (g/day) in 1980, 1986, 2001, and 2007 were combined as the mean long-term intake of SFA which was energy-adjusted using the residual method and divided into thirds by tertile classification for the further analyses.

### Physical Activity

Three sum indices of physical activity collected with the questionnaires in 2007 and 2008 were used to describe the habitual physical activity of the participants and skeletal loadings [31, 36]. In 2007, the items of the physical activity index were the intensity of physical activity, frequency and duration of vigorous physical activity, the average duration of a physical activity session, and participation in organized exercise training. In 2008, the bone loading indices of radius and tibia were collected through the questions concerning the type of mechanical loading exerted on bone and calculated with the following equation for each type of mechanical loading: participant’s body weight (kg) × EL × I x ln (1 + F × D), where EL = exercise loading, I = intensity of exercise (ranging from 1 to 4, where 1 = no breathlessness at all and 4 = strong breathlessness and sweating), F = frequency of exercise (asked in two questions; how many months of the year and how often per month or week), and D = duration of exercise (ranging from 1 to 5, where 1 = under 20 min and 5 = more than 2 h). The total bone loading indices of radius and tibia combined each type of exercise loading which were high impacts (e.g., ball games, track and field events), strength training, repetitions (e.g., jogging, skiing), non-weight-bearing exercise (e.g., cycling, swimming), and work or recreational activities (e.g., working in the garden, construction work). In addition to leisure-time physical activity, physical load of work was estimated with questions regarding if a participant was in sedentary or manual work.

### Clinical Measures

Body weight and height were measured, and body mass index (BMI, kg/m^2^) was calculated. Alcohol consumption was estimated from a self-administered questionnaire as the standard number of drinks per day. Smoking habits were defined as pack-years (the number of years a person has smoked one pack of cigarettes per day) and as a proportion of daily smokers (%). In addition, the use of oral corticosteroid medication for at least one month (yes/no) was gathered with the questionnaire.

Participants’ venous blood samples were drawn after an overnight fast and serum was separated for the biochemical analyses [30]. The levels of serum total cholesterol (mmol/l), high-density lipoprotein (HDL)-cholesterol (mmol/l), and glucose (mmol/l) were measured using enzymatic assays with an AU400-analyser (Olympus, Japan). Low-density lipoprotein (LDL)-cholesterol was estimated using the Friedewald formula in those with triglycerides < 4.0 mmol/l. In addition, due to changes in methods or kits between the years 2001 and 2007, the levels of glucose and insulin were corrected with the specific equations as described elsewhere [37]. Serum calcidiol concentrations (nmol/l) were determined using radioimmunoassay (DiaSorin, Stillwater, Minnesota).

### Statistical Analyses

The analysis of variance, Brown–Forsythe, and Chi-square tests were chosen to compare the participant characteristics in the tertiles of dietary SFA intake after the tests of homogeneity of group variances. Analysis of covariance with the established risk factors of osteoporosis as covariates (age, height, weight, energy intake, serum calcidiol concentration, calcium intake, physical activity in leisure-time and work, and smoking) was then performed to study if the between-group differences in bone phenotypes measured with pQCT and QUS showed a dose-dependent association. The fracture frequencies across the tertiles of dietary SFA intake were tested with the Chi-square test. In addition, the adjusted odds ratios (ORs) of fractures and their 95% confidence intervals (95% CIs) in the tertiles of dietary SFA intake were calculated with binary logistic regression analysis (the lowest intake of SFA as the reference). All comparisons were performed with IBM SPSS Statistics 27 separately for women and men. To avoid possible multiple comparison bias, those associations that replicated in both males and females with P value less than 0.05 were interpreted as being supportive for the main hypothesis.

## Results

### Background Characteristics

Background characteristics of participating women and men within the tertiles of dietary SFA intake are shown in Tables 1 and 2, respectively. Women with the lowest intake of SFA (≤ 27.4 g/day) had 694 kJ higher energy intake per day compared with women in the highest tertile of SFA intake (*P* < 0.001, Table 1). In addition, their diet included more carbohydrates, fiber, calcium, vitamin D, milk, and sour-milk products compared with women in the two upper tertiles of SFA intake (*P* ≤ 0.04, Table 1). The intakes of fats in total, monounsaturated fats, and cheese were, on the contrary, higher among women in the upper tertiles of SFA intake compared with the lowest tertile (*P* < 0.001). Like the intake of dietary vitamin D, also serum calcidiol concentration was the highest among women in the lowest tertile of SFA intake (*P* < 0.001). In addition, there were also fewer daily smokers in the lowest tertile of SFA intake and their physical activity indices were higher compared with the upper tertiles of SFA intake (*P* ≤ 0.02, Table 1).

**Table 1:** Background characteristics of women as mean values (SD) or frequencies (%, n) in the thirds of dietary saturated fat

	Dietary long-term intake of saturated fat, g/day			P value*
	≤ 27.43 (*n* = 333–495)	27.44–35.44 (*n* = 303–495)	≥ 35.45 (*n* = 331–495)	
Age, years	37.8 (5)	37.6 (5)	37.6 (4.9)	0.84
Height, cm	166 (6.1)	165.9 (5.2)	165.9 (6.5)	0.97
Weight, kg	69.8 (13.8)	70.6 (15.7)	69.3 (13.9)	0.52
Body mass index, kg/m^2^	25.3 (4.8)	25.6 (5.4)	25.2 (4.8)	0.55
Energy intake, kJ/day	9635 (2860)	8823 (2732)	8941 (2808)	< 0.001
Carbohydrate, E%	50.4 (5)	46.8 (5.1)	44.5 (5)	< 0.001
Fiber, g/day	29.7 (11.1)	23.3 (9.2)	21.2 (8.1)	< 0.001
Protein, E%	17.4 (2.4)	17.7 (2.4)	17.2 (2.3)	0.007
Fat, E%	29.1 (3.8)	32.4 (3.9)	35.4 (3.9)	< 0.001
Polyunsaturated fat, E%/g/day	5.1/13 (4.7)	5.3/12.4 (4.6)	5.2/12.3 (4.4)	0.10
Monounsaturated fat, E%/g/day	9.8/24.9 (8.6)	10.9/25.5 (9.5)	11.6/27.5 (9.4)	< 0.001
Dietary calcium, mg/day	1419 (532)	1322 (547)	1352 (561)	0.04
Dietary vitamin D, μg/day	7.5 (3)	6.7 (3)	6.1 (2.7)	< 0.001
Intake of milk, g/day	369 (284)	320 (276)	303 (272)	0.003
Intake of sour-milk products, g/day	198 (176)	178 (187)	162 (184)	0.03
Intake of cheese, g/day	36 (25)	41 (33)	49 (32)	< 0.001
Serum calcidiol, nmol/l	64.4 (22.1)	59.8 (20.1)	59.1 (19.2)	< 0.001
Serum glucose, mmol/l	5.2 (1)	5.1 (0.6)	5.2 (0.8)	0.45
Serum insulin, mU/l	8.9 (10.3)	9 (7.9)	8.4 (6.3)	0.55
Serum total cholesterol, mmol/l	4.9 (0.9)	4.9 (0.8)	5.0 (0.9)	0.38
Serum HDL-cholesterol, mmol/l	1.4 (0.3)	1.4 (0.3)	1.5 (0.3)	0.78
Serum LDL-cholesterol, mmol/l	2.9 (0.7)	3 (0.7)	3 (0.8)	0.21
Alcohol consumption, drinks/day	0.5 (0.7)	0.6 (0.7)	0.6 (0.8)	0.13
Current daily smokers, %	9.9 (40 women)	15.5 (59)	20.4 (79)	< 0.001
Oral corticosteroid medication ≥ 1 month, %	7.9 (27 women)	8.1 (26)	4.9 (17)	0.17
Those in manual work, %	25.8 (93 women)	29.2 (98)	31 (104)	0.38
Physical activity index	9.2 (1.7)	8.7 (1.7)	8.6 (1.7)	< 0.001
Radial bone loading index	382 (357)	334 (319)	319 (357)	0.05
Tibial bone loading index	635 (627)	523 (499)	521 (584)	0.02

*Tested with analysis of variance, Brown–Forsythe-test, or Chi-Square test

**Table 2:** Background characteristics of men as mean values (SD) or frequencies (%, n) in the thirds of dietary saturated fat

	Dietary long-term intake of saturated fat, g/day			P value*
	≤ 35.58 (*n* = 246–440)	35.59–48.97 (*n* = 222–442)	≥ 48.98 (*n* = 248–441)	
Age, years	37.4 (4.9)	37 (5.1)	37.8 (4.9)	0.08
Height, cm	179.5 (6.8)	180.3 (6.6)	179.3 (6.4)	0.16
Weight, kg	84.6 (14.4)	87.3 (16.2)	87.3 (15.6)	0.04
Body mass index, kg/m^2^	26.2 (3.9)	26.8 (4.3)	27.1 (4.4)	0.03
Energy intake, kJ/day	11,369 (3101)	10,801 (4260)	11,776 (4419)	0.01
Carbohydrate, E%	47 (5.7)	44 (4.7)	41.9 (5.1)	< 0.001
Fiber, g/day	27.2 (11)	22.3 (10.4)	22 (10.4)	< 0.001
Protein, E%	17.5 (2.7)	17.9 (2.4)	17.4 (2.4)	0.06
Fat, E%	30.2 (4.2)	33.5 (3.6)	37 (4.2)	< 0.001
Polyunsaturated fat, E%/g/day	5.3/15.8 (5.3)	5.4/15.5 (6.3)	5.5/17.1 (7.4)	0.007
Monounsaturated fat, E%/g/day	10.4/31.3 (10.3)	11.6/33 (14.2)	12.6/39.1 (16.3)	< 0.001
Dietary calcium, mg/day	1505 (558)	1490 (669)	1638 (734)	0.01
Dietary vitamin D, μg/day	9.6 (3.8)	8.6 (3.7)	8.8 (4.1)	0.007
Intake of milk, g/day	484 (368)	439 (350)	502 (404)	0.12
Intake of sour-milk products, g/day	148 (149)	140 (180)	143 (172)	0.84
Intake of cheese, g/day	36 (28)	44 (33)	52 (40)	< 0.001
Serum calcidiol, nmol/l	58.1 (16.3)	57.4 (17.6)	54.6 (16.4)	0.02
Serum glucose, mmol/l	5.5 (1.3)	5.5 (0.8)	5.5 (0.5)	0.97
Serum insulin, mU/l	8.5 (6.7)	10.3 (13.0)	11.9 (37.3)	0.19
Serum total cholesterol, mmol/l	5.2 (1)	5.2 (1)	5.2 (0.9)	0.98
Serum HDL-cholesterol, mmol/l	1.2 (0.3)	1.2 (0.3)	1.2 (0.3)	0.77
Serum LDL-cholesterol, mmol/l	3.3 (0.8)	3.3 (0.8)	3.3 (0.8)	0.76
Alcohol consumption, drinks/day	1.5 (2.3)	1.5 (1.6)	1.3 (1.6)	0.26
Current daily smokers, %	15.3 (48 men)	25.4 (76)	27.0 (86)	< 0.001
Oral corticosteroid medication ≥ 1 month, %	1.2 (3 men)	2.2 (5)	4.7 (12)	0.04
Those in manual work, %	33 (96 men)	39.5 (113)	56.9 (174)	< 0.001
Physical activity index	9.2 (1.8)	8.8 (1.8)	8.3 (2)	< 0.001
Radial bone loading index	559 (637)	384 (381)	333 (376)	< 0.001
Tibial bone loading index	797 (910)	585 (566)	561 (658)	< 0.001

*Tested with analysis of variance, Brown–Forsythe-test, or Chi-Square test

Men in the lowest tertile of SFA intake had lower body weight and body mass index compared with men in the upper tertiles of SFA intake (*P* ≤ 0.04, Table 2). In addition, the intakes of carbohydrate, fiber, and vitamin D as well as the calcidiol concentration were the highest among men in the lowest tertile of SFA intake (*P* ≤ 0.02). The intakes of energy, fats, calcium, and cheese were, on the contrary, the highest among men in the third tertile of SFA intake (*P* ≤ 0.01, Table 2). In addition, there were more men who smoked daily and had used corticosteroid medication for at least one month in the highest tertile of SFA intake (*P* ≤ 0.04). Men with higher SFA intake were also more frequently manual workers compared with men in the lowest tertile (*P* < 0.001). Physical activity indices were the highest in men with the lowest intake of SFA (*P* < 0.001, Table 2).

### Dietary Saturated Fat and Bone Outcomes

In the radius, cortical density (mg/cm^3^) and cortical-to-total area ratio at the radial shaft differed significantly across the tertiles of SFA intake in women (P for trend ≤ 0.02, Table 3). Both cortical bone traits were 0.1–0.2% lower in women with the highest SFA intake compared with women with the lowest SFA intake (Table 3). No statistically significant differences were found in other radial traits or any trait in the tibia across the tertiles of SFA intake in either gender (Tables 3 and 4).

**Table 3:** Mean values (SD) of peripheral quantitative computed tomography (pQCT) in the thirds of dietary saturated fat in women

	Dietary long-term intake of saturated fat, g/day			ANCOVA*
pQCT bone traits^a^	≤ 27.43	27.44–35.44	≥ 35.45	P for trend
*Distal radius*	*n* = 252	*n* = 218	*n* = 261	
Total area, mm^2^	315.7 (45.8)	308.8 (39.4)	316 (46.1)	0.57
BMC, mg	201.1 (27.9)	194.1 (26.4)	201.1 (31)	0.27
Trabecular density, mg/cm^3^	208.2 (29.8)	206 (29.4)	208.3 (28.1)	0.14
*Radial shaft*	*n* = 252	*n* = 218	*n* = 261	
Total area, mm^2^	94.1 (13.5)	92.4 (12.4)	94.5 (14.6)	0.56
Cortical area, mm^2^	75.5 (8.2)	74.1 (7.8)	75.7 (8.7)	0.29
Cortical density, mg/cm^3^	1212 (18.5)	1212 (19.7)	1210 (21.3)	0.01
BMC, mg	183 (19.9)	179.7 (19)	183.2 (20.8)	0.13
Cortical-to-total area ratio	0.809 (0.06)	0.808 (0.06)	0.808 (0.06)	0.02
SSI, mm^3^	206.8 (38.1)	203 (36.7)	208.5 (42.3)	0.95
*Distal tibia*	*n* = 255	*n* = 220	*n* = 261	
Total area, mm^2^	821.1 (112)	807 (96.9)	817.9 (109.9)	0.79
BMC, mg	523.9 (69)	518.8 (72.7)	521.6 (70.5)	0.10
Trabecular density, mg/cm^3^	229.5 (30.3)	230.5 (31.5)	226.5 (28.7)	0.26
*Tibial shaft*	*n* = 255	*n* = 220	*n* = 261	
Total area, mm^2^	341.9 (43.8)	340.2 (40.9)	345.1 (44.1)	0.77
Cortical area, mm^2^	247.2 (30.5)	245.7 (29.1)	249.3 (31.9)	0.23
Cortical density, mg/cm^3^	1170 (19.9)	1168 (19.6)	1169 (18.7)	0.15
BMC, mg	577.9 (68.2)	574 (67.1)	582.3 (72.5)	0.15
Cortical-to-total area ratio	0.725 (0.05)	0.724 (0.05)	0.724 (0.05)	0.15
SSI, mm^3^	1304 (237.1)	1292 (221.3)	1326 (245.4)	0.50

^a^BMC, bone mineral content; SSI, stress–strain index

*Analysis of covariance (ANCOVA) included age, height, weight, energy intake, serum calcidiol concentration, calcium intake, physical activity, physical load of work, and smoking as covariates

**Table 4:** Mean values (SD) of peripheral quantitative computed tomography (pQCT) in the thirds of dietary saturated fat in men

	Dietary long-term intake of saturated fat, g/day			ANCOVA*
pQCT bone traits^a^	≤ 35.58	35.59–48.97	≥ 48.98	P for trend
*Distal radius*	*n* = 197	*n* = 169	*n* = 187	
Total area, mm^2^	408.8 (61.7)	415.3 (62.3)	428.9 (64.3)	0.84
BMC, mg	296.8 (42)	300.8 (43.9)	313.4 (43.1)	0.58
Trabecular density, mg/cm^3^	248.6 (30.6)	247.1 (32.8)	250.5 (30.1)	0.22
*Radial shaft*	*n* = 197	*n* = 169	*n* = 187	
Total area, mm^2^	134.6 (18.1)	138.2 (19.4)	144 (18.2)	0.63
Cortical area, mm^2^	106.3 (12.4)	107.8 (13)	111.9 (12.6)	0.62
Cortical density, mg/cm^3^	1185 (22.9)	1180 (21.5)	1179 (22.2)	0.21
BMC, mg	251.8 (29.1)	254.4 (29.8)	263.8 (29.3)	0.47
Cortical-to-total area ratio	0.794 (0.05)	0.784 (0.06)	0.780 (0.05)	0.97
SSI, mm^3^	332.9 (62.4)	342.9 (61.4)	362.6 (63.8)	0.47
*Distal tibia*	*n* = 196	*n* = 166	*n* = 194	
Total area, mm^2^	977.3 (128.4)	994.9 (141.6)	989.4 (136)	0.90
BMC, mg	702.9 (103.1)	702.6 (104.3)	704.2 (101.1)	0.52
Trabecular density, mg/cm^3^	257.3 (34)	254.9 (34.9)	254.9 (32.5)	0.31
*Tibial shaft*	*n* = 196	*n* = 166	*n* = 194	
Total area, mm^2^	430.3 (51.5)	438.2 (51.4)	438.2 (49.3)	0.96
Cortical area, mm^2^	319.5 (42.8)	318.9 (39.7)	319.7 (40.2)	0.71
Cortical density, mg/cm^3^	1148 (21.6)	1146 (22.9)	1147 (22.1)	0.58
BMC, mg	732.8 (95)	730.1 (85.8)	732.8 (88)	0.63
Cortical-to-total area ratio	0.743 (0.05)	0.729 (0.05)	0.731 (0.05)	0.54
SSI, mm^3^	1806 (319)	1841 (301.3)	1836 (295.8)	0.94

^a^BMC, bone mineral content; SSI, stress–strain index

*Analysis of covariance (ANCOVA) included age, height, weight, energy intake, serum calcidiol concentration, calcium intake, physical activity, physical load of work, and smoking as covariates

At the calcaneus, difference in SOS value (m/s) across the tertiles of SFA intake was statistically significant only in women (*P* = 0.04, Table 5). Women with the highest intake of SFA had 0.4% lower SOS value compared with women with the lowest SFA intake (Table 5).

**Table 5:** Mean values (SD) of quantitative ultrasound bone measurements at calcaneus in the thirds of dietary saturated fat

	Dietary long-term intake of saturated fat, g/day			ANCOVA*
Women	≤ 27.43 (*n* = 226)	27.44–35.44 (*n* = 179)	≥ 35.45 (*n* = 211)	P for trend
BUA^a^, dB/MHz	81.4 (15.2)	77.2 (16.8)	77.7 (13)	0.58
SOS^a^, m/s	1563 (28)	1555 (31.5)	1557 (25.7)	0.04
Men	≤ 35.58 (*n* = 155)	35.59–48.97 (*n* = 130)	≥ 48.98 (*n* = 147)	
BUA^a^, dB/MHz	82 (15.9)	82.2 (16.1)	80.7 (15.8)	0.62
SOS^a^, m/s	1561 (27.4)	1558 (27.5)	1557 (29.1)	0.38

^a^BUA, broadband ultrasound attenuation; SOS, speed of sound

*Analysis of covariance (ANCOVA) included age, height, weight, energy intake, serum calcidiol concentration, calcium intake, physical activity, physical load of work, and smoking as covariates

In addition, there were 60, 75, and 87 men who had at least one fracture in the first, second, and third tertile of SFA intake, respectively (Table 6, χ^2^-test, *P* = 0.05). The most common fracture sites in the present population were shoulder area and upper arm, forearm, and lower leg (Table 6). Also, the adjusted odds ratio for having at least one fracture was increased in men in the second (OR 1.86, 95% CI 1.03–3.33, *P* = 0.04) and third tertile of SFA intake (OR 2.45, 95% CI 1.38–4.36, *P* = 0.002) compared with men with the lowest SFA intake. However, no significant association of fractures and SFA intake was found in women (ORs 1.10–1.25, 95% CI 0.59–2.28, *P* = 0.46–0.76).

**Table 6:** Fracture frequencies (%, n) in the thirds of dietary saturated fat

	Mean dietary long-term intake of saturated fat, g/day (SD)			P value*
	22 (4.2) *n* = 495	31.3 (2.3) *n* = 495	44.2 (11.2) *n* = 495	
Women with previous fracture(s), %	8.1 (40 women)	6.7 (33)	8.3 (41)	0.62
Of whom with lumbar spine or pelvis fracture, %	0.6 (3)	0.2 (1)	0 (0)	0.33
Of whom with skull or facial bone fracture, %	0.4 (2)	0 (0)	0.2 (1)	0.78
Of whom with cervical spine fracture, %	0.2 (1)	0 (0)	0 (0)	1.0
Of whom with thoracic fracture, %	0.8 (4)	0.6 (3)	0.8 (4)	1.0
Of whom with shoulder or upper arm fracture, %	0.8 (4)	1.2 (6)	1.6 (8)	0.56
Of whom with forearm fracture, %	2 (10)	0.8 (4)	2.4 (12)	0.13
Of whom with wrist or hand fracture, %	0.4 (2)	1.2 (6)	0.6 (3)	0.41
Of whom with femoral fracture, %	0.4 (2)	0 (0)	0.4 (2)	0.56
Of whom with lower leg fracture, %	3 (15)	2.2 (11)	3 (15)	0.69
Of whom with foot fracture, %	0.4 (2)	0.6 (3)	0 (0)	0.38

	27.8 (5.6) *n* = 440	41.9 (3.7) *n* = 442	63.3 (15.6) *n* = 441	
Men with previous fracture(s), %	13.6 (60 men)	17.0 (75)	19.7 (87)	0.05
Of whom with lumbar spine or pelvis fracture, %	0.7 (3)	0.2 (1)	1.1 (5)	0.24
Of whom with skull or facial bone fracture, %	2.3 (10)	3.2 (14)	3.2 (14)	0.67
Of whom with cervical spine fracture, %	0.2 (1)	0.2 (1)	0.5 (2)	0.85
Of whom with thoracic fracture, %	1.4 (6)	1.4 (6)	1.1 (5)	0.96
Of whom with shoulder or upper arm fracture, %	2.7 (12)	2.7 (12)	3.6 (16)	0.67
Of whom with forearm fracture, %	1.1 (5)	2.3 (10)	4.1 (18)	0.02
Of whom with wrist or hand fracture, %	1.8 (8)	1.4 (6)	2 (9)	0.70
Of whom with femoral fracture, %	1.4 (6)	1.8 (8)	1.8 (8)	0.89
Of whom with lower leg fracture, %	4.3 (19)	6.6 (29)	5.2 (23)	0.34
Of whom with foot fracture, %	0.5 (2)	1.4 (6)	1.4 (6)	0.34

*Tested with Chi-Square test

## Discussion

In the present population-based study, we found no consistent associations between SFA intake and bone health indicators. In the previous studies, saturated fat has been found to enhance bone resorption rather than bone formation and has an unfavorable impact on cortical density at the femur and the risk of hip fractures [3, 11–18]. Since few human studies are done on this topic previously, only tentative conclusions can be made of the associations of SFA and bone in humans. It may be, for instance, that the association of SFA and bone differs according to each bone phenotype or other factors may influence this association. In the present study, the dietary intake of vitamin D and physical activity indices were greater in both genders, and the frequency of daily smoking lower in the lowest tertile of SFA intake compared with those with higher SFA intake. In men, those in the highest tertile of SFA intake had also higher body weight and greater intake of calcium compared with the lowest tertile. In addition, in men, difference was seen in socioeconomic occupational status as those with higher SFA intake worked more frequently in manual work. In the previous studies of the Young Finns cohort, overweight [31, 38], the dietary intake of calcium and leisure-time physical activity [39–41], and exposure to passive smoke in childhood [42] have been found to be associated with different bone outcomes in the present population. Therefore, all these generally well-known risk factors of osteoporosis were included in the statistical model to exclude their possible confounding effect on the results. Also, other nutritional and clinical factors were considered as potential confounding factors between SFA intake and bone phenotypes but were not included in the model because of correlations with factors already existing in the model, or there was no adequate previous evidence of bone effect. For instance, the intake of protein correlated with total energy intake which was added to the final model. In addition, the differences in protein intake are clinically small although statistically significant in women. All the participants had also enough protein in their diets if compared with the national nutritional recommendation which is 10–20% of total energy intake. Greater differences were seen in the intakes of carbohydrate and fat, but as these energy nutrients are less studied relative to bone health, we decided not to add them to the final model.

The strength of the present study was that both a variety of bone phenotypes and fractures were drawn from a multi-center study including both women and men. In addition, lifestyle information on the Young Finns cohort has been gathered since 1980 which have yielded a longitudinal data, among other things, of dietary SFA intake. Although, two different dietary methods were used to study the intake of SFA over the study years, we assume that this has not considerably confounded the results as the mean intake of SFA was calculated and energy-adjusted and further divided into three equal-sized groups for the statistical comparisons. This should have given comparable information on the long-term SFA intake of the present cohort. There are, however, some shortcomings with the used dietary methods as the information gathered is self-reported. Thus, it is probable that reporting bias occurs as it may be difficult to recall all the foods and beverages that one has actually consumed. Some of the participants may therefore have exaggerated their eating habits, whereas others may have reported smaller portions of foods and beverages than usual. However, in general, underreporting and overreporting are common features, not only in nutrition research, but also in other areas of research that examine lifestyle habits in humans.

In addition, there may be some other reasons for small differences in bone phenotypes between the SFA tertiles of present population. Surprisingly, no differences were seen in blood cholesterol and glucose levels between the SFA groups which earlier have related to the function of bone cells and bone marrow metabolism [4, 5, 19]. In addition, it might be that the differences in the intake of dietary saturated fat were too small between the SFA tertiles that any remarkable differences in bone phenotypes could be detected. This explanation is supported by the fact that according to the latest national survey, most adult Finns consume more saturated fat than is recommended [43]. Thus, it may be difficult to find study participants who consume the recommendable amount of saturated fat. In men, the dietary intake of saturated fat was, however, associated with the incidence of all fractures occurred between 1980 and 2018. Men also had the greater intake of saturated fat compared with women which may partly explain why the association of SFA and fractures was found only in men. Fractures were gathered from the national health care registers and hence confirmed by the healthcare professionals in Finnish hospitals. In general, bone fractures have clinical and economic significance when considering the harm they cause to the individual and more broadly [44]. However, no association of dietary saturated fat and fractures was found in women, why further studies would be needed to confirm if these fractures really are associated with SFA intake or, for instance, with accidents at work and leisure-time activities. Specific reasons how the fractures occurred remain still uncertain as no information was available on this matter in registered data.

## Conclusion

We did not find consistent associations between bone phenotypes and SFA intake that would have replicated in both men and women. Therefore, large clinical and prospective studies are needed to confirm if dietary SFA intake affects bone health in humans.
